# Clinicopathological and molecular characteristics of colorectal adenosquamous carcinoma in an Asian population

**DOI:** 10.1186/s12876-023-02989-9

**Published:** 2024-01-16

**Authors:** Fujin Ye, Mian Chen, Xiaobin Zheng, Pinzhu Huang, Chao Wang, Huashan Liu, Hao Xie, Wei Xiao, Qin Guo, Liang Huang

**Affiliations:** 1 Department of General Surgery (Colorectal Surgery), The Sixth Affiliated Hospital, Sun Yat-sen University, Guangzhou, China; 2 Guangdong Provincial Key Laboratory of Colorectal and Pelvic Floor Diseases, The Sixth Affiliated Hospital, Sun Yat-sen University, Guangzhou, China; 3 Department of Medical Oncology, The Sixth Affiliated Hospital of Sun Yat-sen University, Guangzhou, 510655 Guangdong China; 4 Department of Pathology, The Sixth Affiliated Hospital of Sun Yat-sen University, Guangzhou, 510655 Guangdong China

**Keywords:** Adenosquamous carcinoma, *KRAS* mutation, Mismatch repair-deficient

## Abstract

**Background:**

Adenosquamous carcinoma is a rare sub-type of colorectal cancer with a poor prognosis. Little is known about its clinicopathological and molecular characteristics in Asian populations. This study aimed to investigate these features in a cohort of patients with adenosquamous carcinoma in the colorectum.

**Methods:**

Tumor cases pathologically diagnosed with colorectal adenosquamous carcinoma were retrieved from the Sixth Affiliated Hospital, Sun Yat-sen University tissue archive between December 2012 and June 2020. Clinicopathological features, molecular characteristics, and oncology outcomes were analyzed.

**Results:**

Among 18,139 cases of colorectal cancer, 11 were diagnosed with adenosquamous carcinoma, providing an incidence rate of 0.061%. The median overall survival (OS) was 14 months, and the expected 3-year OS rate was 29.6%. As of October 14, 2022, four cases had local recurrence and five had distant metastasis. *KRAS* gene mutations were found in four of seven patients (57.1%), and three out of eleven (27.3%) patients had mismatch repair-deficient (dMMR) tumors.

**Conclusions:**

Adenosquamous carcinoma is associated with a poor prognosis. Compared to other sub-types of colorectal cancer, a higher proportion of patients with dMMR and *KRAS* mutations were observed. These findings suggested that more patients with adenosquamous carcinoma could benefit from targeted therapies, such as immunotherapy.

## Background

Colorectal cancer is a significant medical and economic burden worldwide, particularly in developed countries [1]. Adenosquamous carcinoma is a rare subtype of colorectal cancer that accounts for only 0.06-0.18% of tumor incidence and has a poor prognosis [2]. This subtype contains both adenocarcinoma and squamous carcinoma components. Compared to adenocarcinoma, colorectal adenosquamous carcinoma (CASC) has a worse prognosis and is primarily treated with surgery [3].

Since CASC tumors are rare, most studies have been retrospective analyses. The most established retrospective studies have been database-based studies, such as the first NCI SEER database study, which found that patients with adenosquamous carcinoma had poor survival [4]. Another study by Masoomi found higher overall and colorectal-specific mortality of CASC compared with adenocarcinoma [3]. The latest database study based on the SEER cohort concluded that adenosquamous carcinoma tends to present with an advanced stage, poor differentiation, and poor overall survival (OS) [5]. However, studies based on Asian populations have been limited, and the molecular characteristics of CASC have seldom been mentioned in previous literature [6].

In the present study, we analyzed the molecular characteristics (*KRAS*, *BRAF*, and *PIK3CA* mutations, and the status of MMR) of CASC in Asian patients. Additionally, we investigated the clinicopathological characteristics of CASC patients by conducting a single-center cohort study with detailed follow-up information.

## Materials and methods

### Patients and clinical information

This retrospective, single-center study analyzed 11 patients with adenosquamous carcinoma from The Sixth Affiliated Hospital, Sun Yat-sen University, Guangzhou, China, identified between 14 and 2011 and 14 October 2020. Demographic characteristics, preoperative imaging, surgical procedures, preoperative and postoperative treatments, and other related information were collected.

### Follow-up information

Follow-up started on the date of surgery and continued until October 14, 2022, with visits scheduled every 3 months during the first year, biannually from years 1 to 5, and annually after 5 years. Follow-up methods included a re-examination of outpatients and inpatients with medical records and telephone follow-ups. Imagine detection including CT (computed tomography), MRI (magnetic resonance imaging), pathological biopsy findings, and other collected data were used to define recurrence and metastasis. During the telephone follow-up, the patient’s family informed death, and the date of death was recorded according to the information provided by the receipt of a death certificate from the hospital. All the work was assisted by the Oncology Follow-up Center of The Sixth Affiliated Hospital, Sun Yat-sen University.

### Pathology assessment

**Fig. 1:**
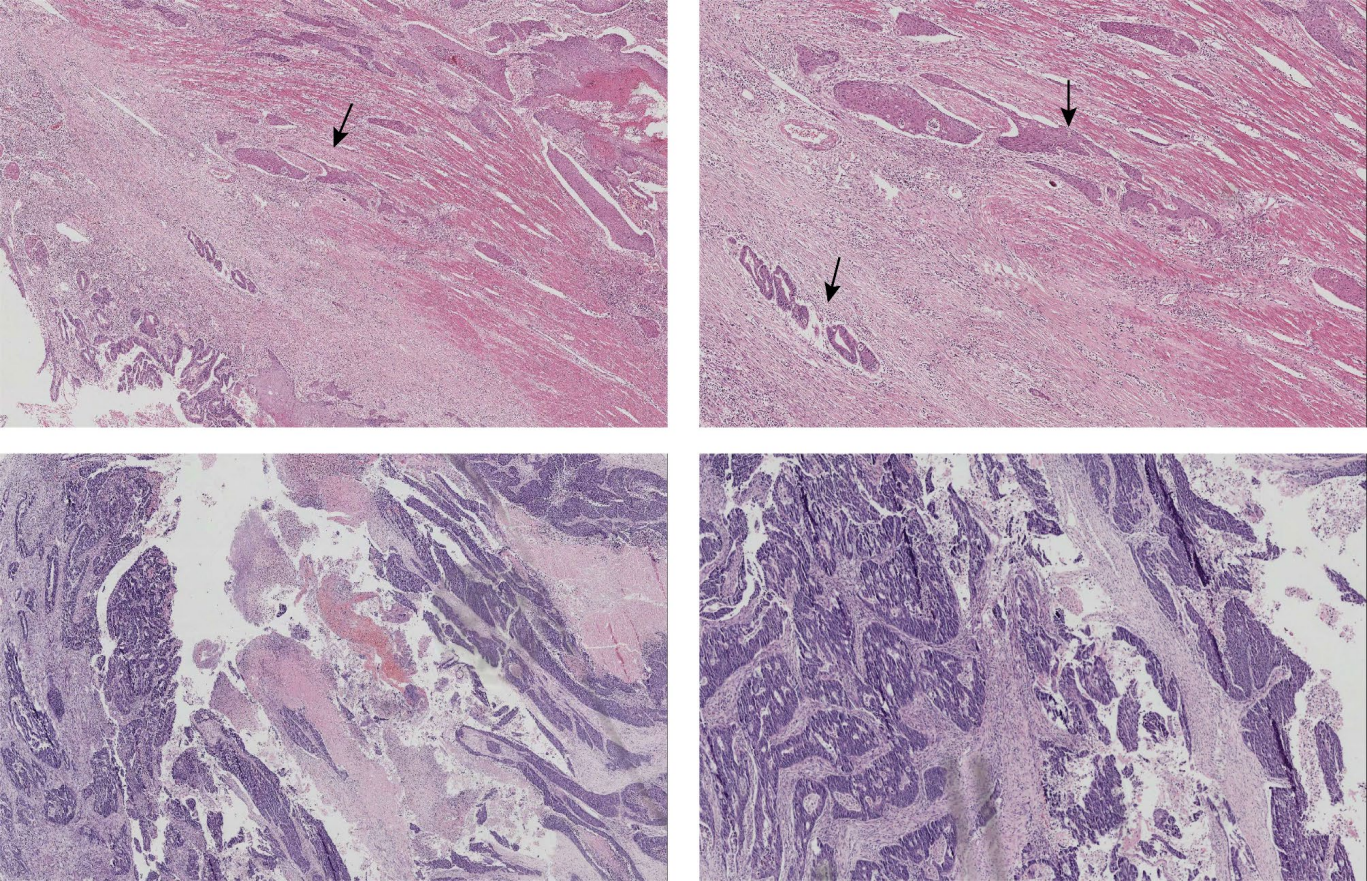
Pathology assessment (H&E staining) Top: post-surgery H&E stain of the sigmoid colon tumor resection specimen from a 59-year-old man with a stage IVc / T4bN2bM1c. The tumor tissue (arrowheads) consisted of both an adenocarcinoma component (10%) and a squamous cell carcinoma component (90%) Bottom: pathological response after neoadjuvant chemotherapy in a 59-year-old woman with stage IIB / T4bN0M0 - residual tissue contained mid-differentiated adenocarcinoma and mid-differentiated squamous cell carcinoma

Individual cases were identified via surgical indices subjected to conventional processing. Two experienced pathologists individually reviewed specimens from CASC patients. Microscopically, the cancerous tissue contained both squamous cells and gland-like cells, which exhibited an irregular glandular duct-like and pore-like arrangement (Fig. 1). Either squamous carcinoma or adenocarcinoma accounts for at least 10% of the total tumor. predominantly squamous type of CASC refers to the squamous component accounting for 60% or more of the total tumor tissues. The patients were staged according to the American Joint Committee on Cancer (AJCC) staging (eighth edition)/International Union Against Cancer (UICC) TNM staging.

### Immunohistochemistry (IHC) for MMR status

**Fig. 2:**
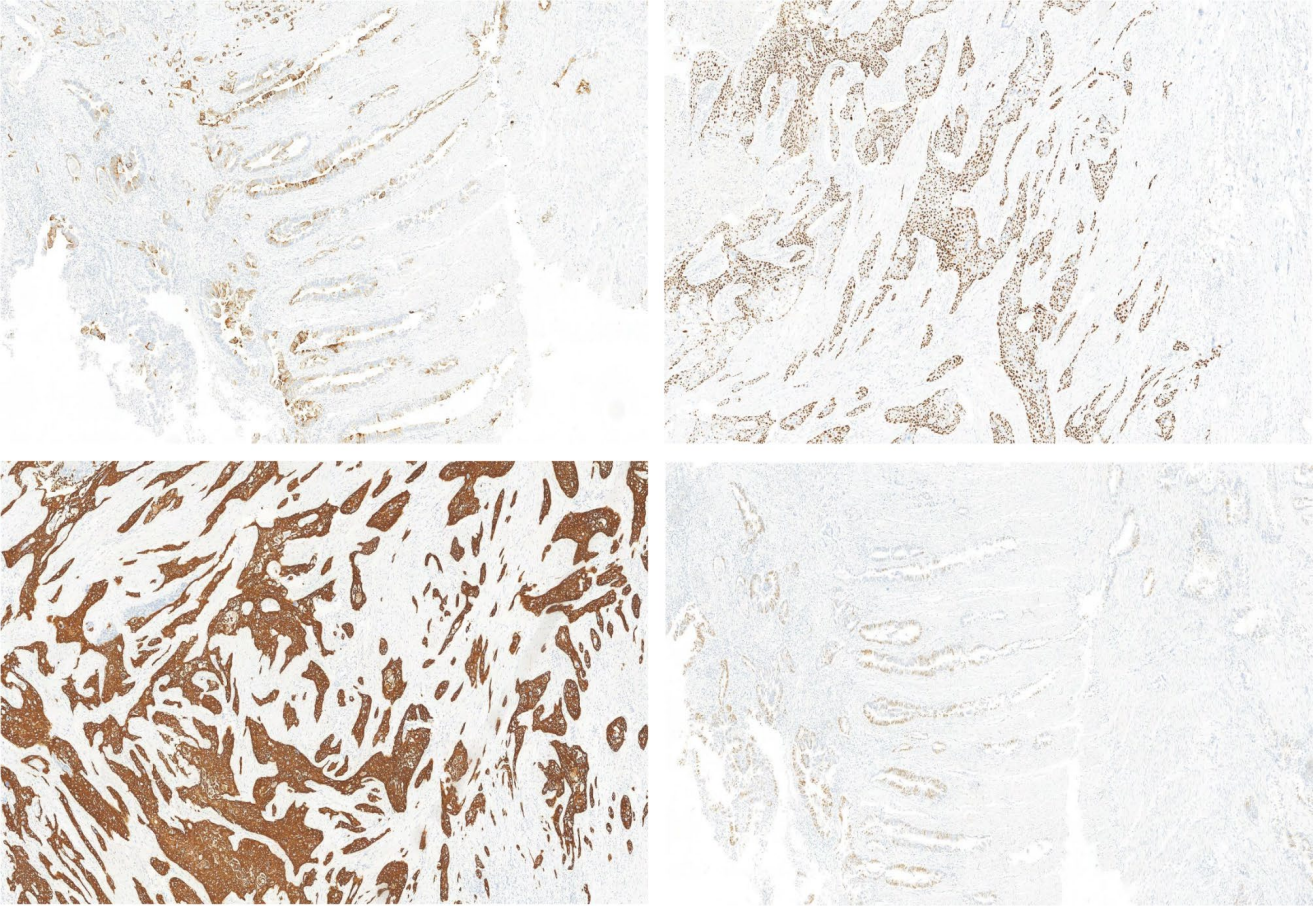
Post-surgery immunostained images from a 22-year-old man with stage I/cT1N0M0. Squamous cell carcinoma markers: CK5/6 (top left), p40 (top right); adenocarcinoma markers: CK20 (bottom left), CDX2(bottom right)

**Fig. 3:**
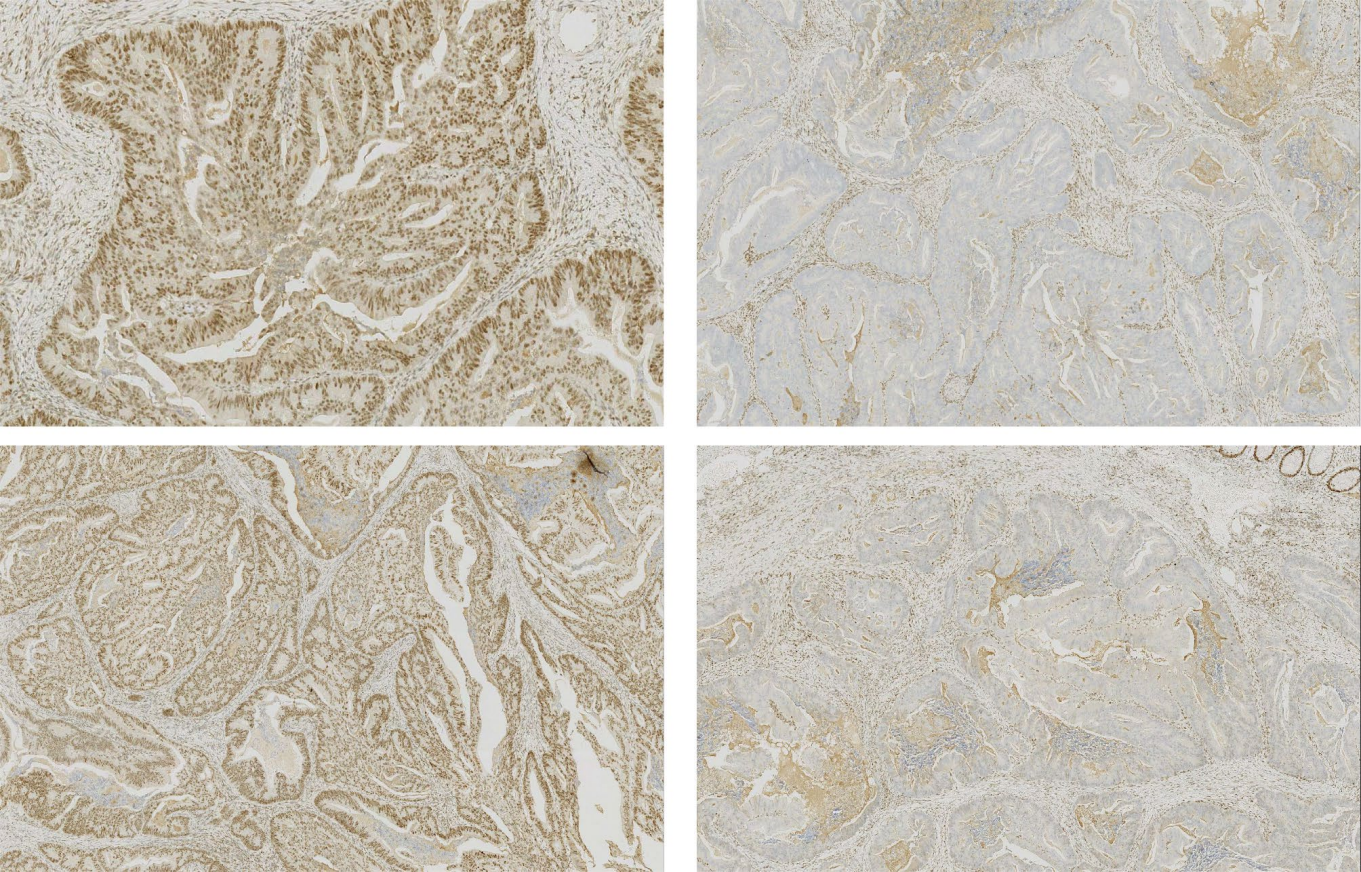
Post-surgery IHC staining from a 22-year-old man with stage I / T1N0M0. Top left: MLH1(+); top right: MSH2(-); bottom left: PMS2(-); bottom right: MSH6(+).

Immunohistochemistry (IHC) was mainly used to make a definitive diagnosis and analyze MMR status. Antigens retrieved from silanized glass slides consist of tumor tissues. The sections were treated with the primary antibodies diluted in the background-reducing solution for CK5/6, p40, CK20, CDX2, and Ki-67. All negative control reactions were phosphate-buffered saline (PBS). Tumor makers for both components (Fig. 2): squamous cell carcinoma markers (CK5/6, p40) and adenocarcinoma markers (CK20, CDX2); MMR status: when there was a complete absence of nuclear staining in tumor cells, tumors were considered negative for MMR proteins (MLH1, MSH2, PMS2, or MSH6). Tumors that maintained expression of all MMR proteins were considered pMMR, otherwise, tumors were considered dMMR (Fig. 3). MMR status would be further confirmed by PCR-based MSI testing if the IHC result was uncertain,

**Fig. 4:**
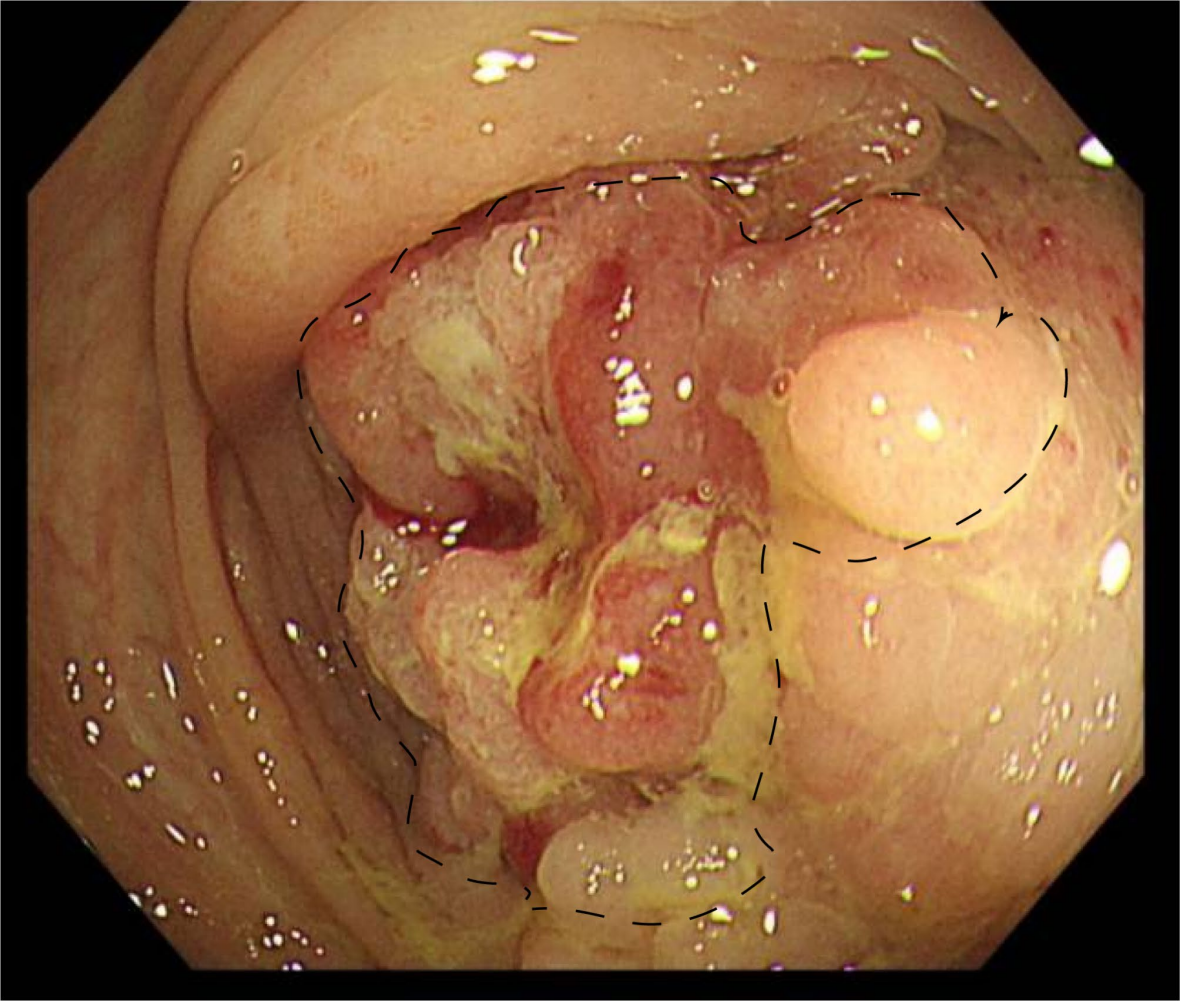
Images of preoperative colonoscopy from a 64-year-old woman with stage IVa / T4aN0M1a. Appropriate dotted lines depicted the extent of the tumor, which was diagnosed as adenosquamous carcinoma on subsequent colonoscopic pathology

**Fig. 5:**
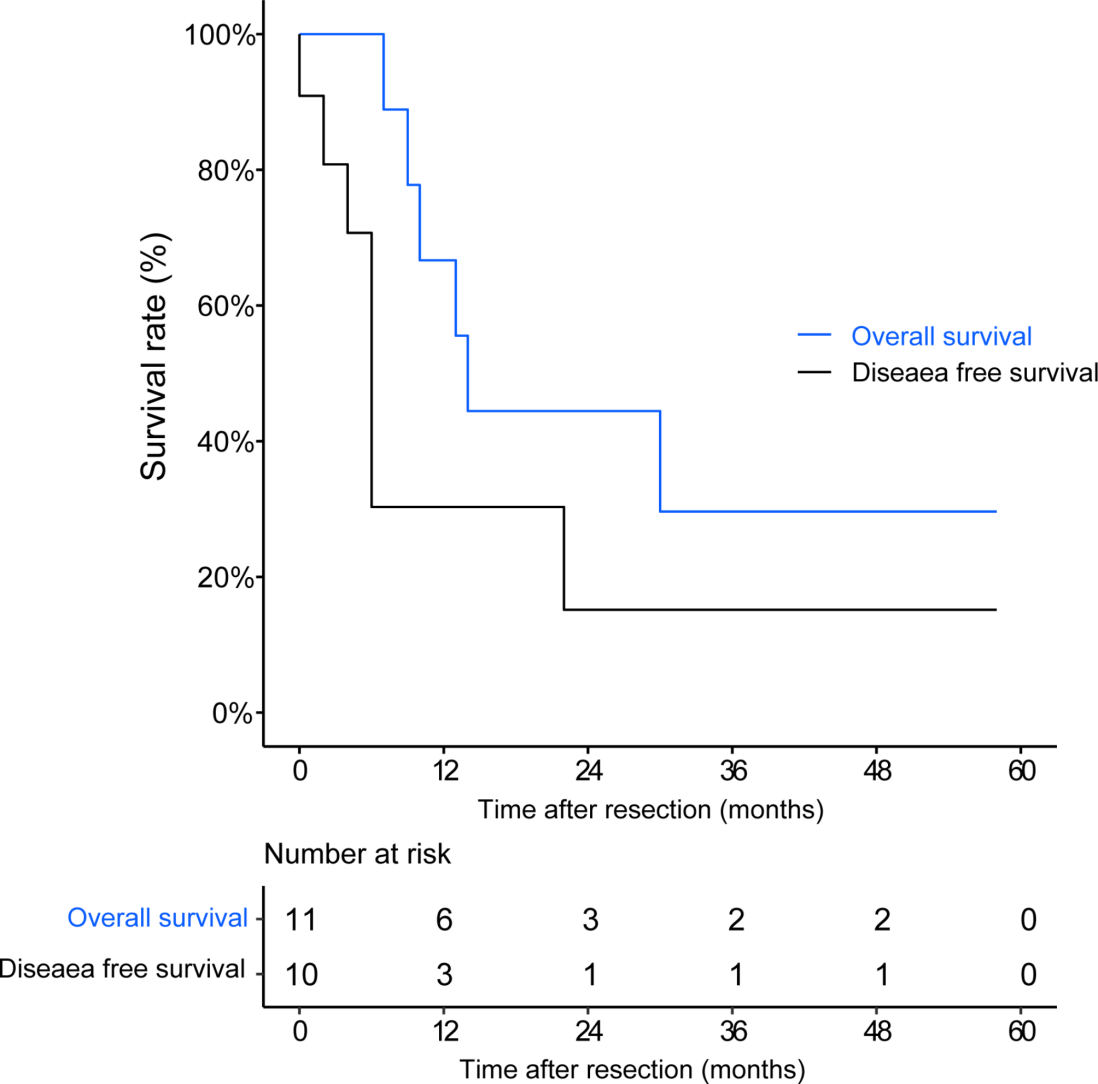
Oncological outcomes in patients with adenosquamous carcinoma

### KRAS, NRAS, BRAF, and PIK3CA mutation analysis

The assessments of *KRAS, BRAF*, and *PIK3CA* mutations were conducted by an adequate quality-control procedure in the Molecular Diagnostic Laboratory of the Sixth Affiliated Hospital of Sun Yat-sen University. Exon 2 (codon 12 and 13), exon 3 and exon 4 of *KRAS*, exon 2 (G12D) and exon 3 (Q61R/K) of *NRAS*, exon 9 (codon 542 and 545) and exon 20 (codon 1047) of *PIK3CA*, and exon 15 (codon 600) of *BRAF* were assessed. An ABI 9700 PCR system was used to conduct PCR amplification. Amplification was done in a 20µL reaction containing 50-100ng of DNA template and 500nM primers, with the following program: 5 min at 98 °C for initial denaturation followed by 45 cycles of 25 s at 95 °C, 25 s at 58 °C, and 25 s at 72 °C, and a final extension at 72 °C for 10 min. PCR products were purified and sequenced by using BigDye Terminator v3.1 Sequencing Standard Kit (Thermo Fisher Scientific, USA) with an ABI Prism 3500Dx Genetic Analyzer (Applied Biosystems, Foster City, CA).

### Statistical analysis

The primary outcomes of this study were overall survival (OS) and disease-free survival (DFS). DFS was defined as the time from surgery to local or systemic recurrence or death, while OS was defined as the time from surgery to death from any cause. The survival curves for OS and DFS were estimated by the Kaplan-Meier method.

The reporting of this study conforms to the STROBE statement [7].

## Results

### Clinicopathological features

Out of the 18,139 patients diagnosed with colorectal cancer during the 10 years from 2011 to 2020, 11 patients were identified with colorectal adenosquamous carcinoma, a prevalence of 0.061% (Table 1). The tumors were predominately located on the left side (63.6%), with 8 cases in the colon and three cases in the rectum. The majority of patients were in an advanced clinical stage, with four cases (36.4%) classified as stage III and five cases (45.5%) as stage IV. The median tumor diameter was 6 cm, and only one case of adenosquamous carcinoma was detected through preoperative colonoscopy (Fig. 4). Microscopic examination revealed a predominantly squamous type (squamous cell carcinoma was the main tumor component) in six patients. Poorly differentiated components were seen in only two patients and the rest presented well or moderately differentiated regions of both components. Lymphovascular invasion was exhibited in two patients and Perineural invasion was in three, other histopathological characteristics: microabscess formation [8] was observed in four patients, tumor necrosis [9] was found in two cases and acellular mucin pools [10] were detected in one patient. Lymph node metastasis was noted in seven cases (63.64%). One patient (male, 22y, T1N0M0) had a family history of polyposis, and another patient (female, 64y, T4aN0M0) had a history of teratoma.

**Table 1:** Clinicopathological features of CASC patients in our cohort

Clinical information	N (%) / Median [IQR]
Age [years]	53[5.5]
Male gender	8(72.7)
Tumor stage	
I	1(9.1)
II	1(9.1)
III	4(36.4)
IV	5(45.5)
Tumor depth	
T1	1(9.1)
T2	1(9.1)
T3	3(27.3)
T4	6(54.5)
Tumor locations	
ileocecal	1(9.1)
Ascending colon	2(18.2)
Transverse colon	1(9.1)
Descending colon	1(9.1)
Sigmoid colon	2(18.2)
Rectum	3(27.3)
Multi-site (sigmoid colon + cecum)	1(9.1)
Ki-67 (%)	40% [15.5%]
Tumor diameter (cm)	6.0 [3.5]
Metastatic sites	
Lung	1(9.1)
Liver	1(9.1)
Peritoneal	2(18.2)
Ureter	1(9.1)
Lymph node	7(63.6)
Neoadjuvant therapy	1(9.1)
Adjuvant therapy	
Chemotherapy	3(27.3)
Immunotherapy	1(9.1)
Radiotherapy	1(9.1)
Tumor progression	7(63.6)
Local recurrence	4(36.4)
Distant metastasis	
Liver	1(9.1)
Ureter	1(9.1)

Ki-67: The tumor cell nuclear proliferation index

### Molecular characteristics

Out of the 11 patients, eight were found to have pMMR (72.7%) and three had dMMR (27.3%). Only one patient was detected as Her-2 positive. The tumor cell nuclear proliferation index Ki-67 ranged from 25 to 70%, with a median of 40%. Among the seven patients who were tested for gene mutations: *KRAS* gene mutations were detected in four patients (57.1%). No mutations were detected in the *NRAS* gene, *BRAF* gene, and *PIK3CA* gene (Table 2).

**Table 2:** The details of gene mutation in our cohort

Case	Gene mutation
M, 46y, IVa / T3N1bM1a	*KRAS* gene exon 2 mutation (Codon 12 is a GGT > GAT mutant)
M, 52y, IVc / T3N1cM1c	*KRAS* gene exon 2 mutation (Codon 1 is a GGC > GAC mutant)
M, 22y, I / T1N0M0	*KRAS* gene exon 4 mutation
F, 64y, IVa / T4aN0M1a	*KRAS* gene exon 3 mutation

M:male F:female

### Oncological outcomes and prognostic analysis

**Fig. 6:**
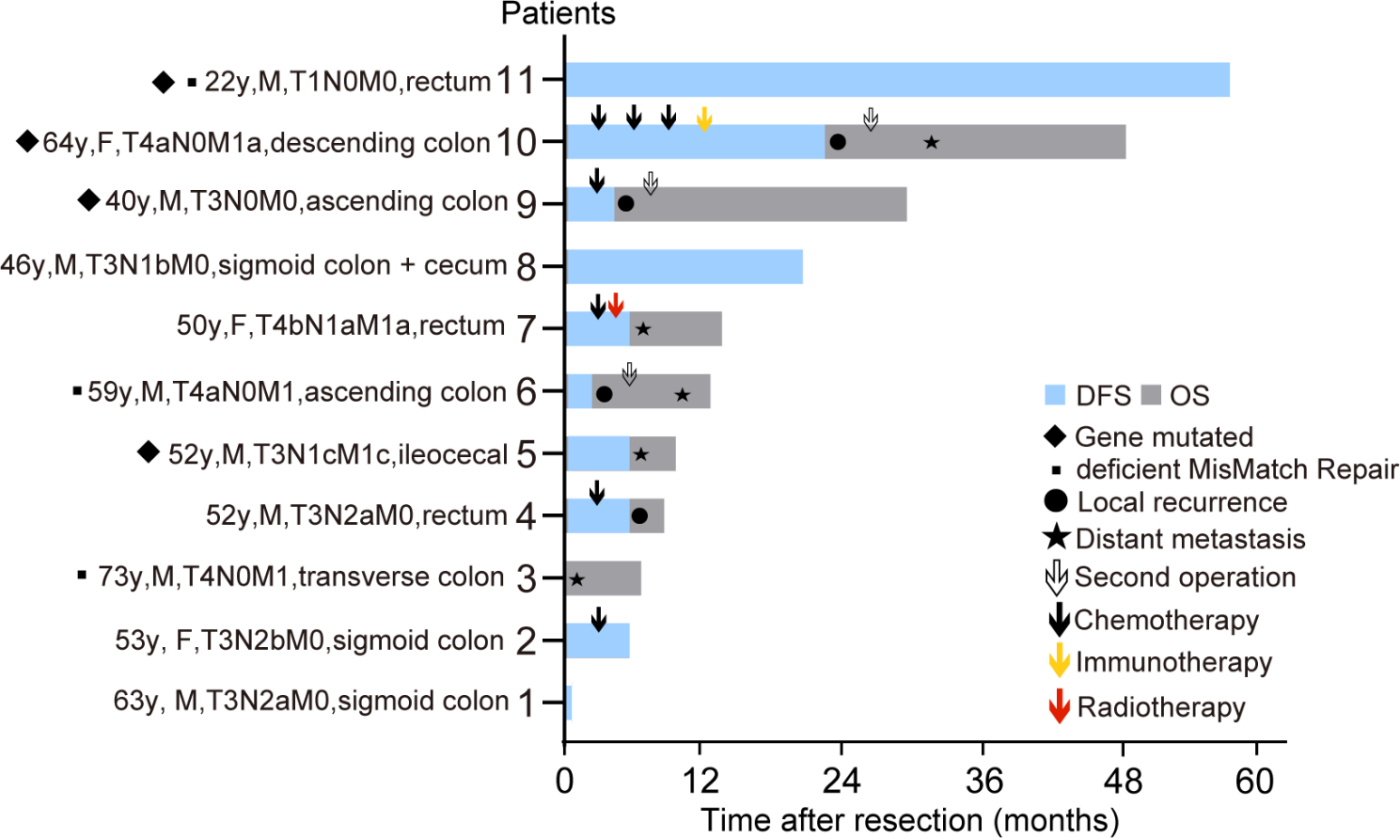
Duration of survival. DFS for disease-free survival, OS for overall survival. M for male, F for female. Arabic numerals plus y represent age, e.g., 73y for 73 years old

As of 14 October 2022, one case was lost to follow-up, three were alive, and seven had died. The median overall survival (OS) period was 14 months (Fig. 5), and the expected 3-year overall survival rate was 29.6%. Local recurrence and distant metastasis were observed in four (Fig. 6) and five cases (one developed distant metastasis preoperatively and two developed distant metastases after local recurrence), respectively, and three cases achieved disease-free survival. The median disease-free survival (DFS) period was 6 months. Among the 11 cases, six were treated with surgery alone, three underwent a second surgery, five received adjuvant chemotherapy (2–8 cycles), one case received adjuvant radiotherapy, and one had neoadjuvant chemotherapy plus immunotherapy (Fig. 6).

## Discussion

The incidence rate of colorectal adenosquamous carcinoma (CASC) in this study was 0.061%, which is consistent with previous reports [2–4]. Compared to adenocarcinoma, CASC patients demonstrated a poorer prognosis. This study is the first to report on the molecular characteristics of CASC patients, including a higher proportion of patients with dMMR (27.3%) and the discovery of *KRAS*-gene mutations in four patients.

The incidence data of CASC has been derived from population-based research, including Cagir’s study [4], the earliest database study that determined an adenosquamous carcinoma incidence rate of 0.06%. Moreover, Masoomi et al. showed an adenosquamous cell carcinoma incidence of 0.09% in 111,263 patients with colorectal adenocarcinoma or adenosquamous carcinoma [3]. Further, the Roswell Park Cancer Institute showed that adenosquamous cell carcinoma accounted for 0.18% of colorectal tumors [11]. According to the latest database study, adenosquamous cell carcinoma accounts for 0.06% of colorectal tumors [2], and an article by Nasseri cites the incidence of adenosquamous carcinoma of the colon as 0.025–0.1% [5]. The CASC incidence derived from our study was consistent with these studies. However, our study is the first to research the incidence of CASC in an Asian population.

The clinicopathological features of patients with adenosquamous carcinoma of the colorectum include a higher incidence in the elderly population [2]. Clinical presentations are dominated by non-specific symptoms such as abdominal pain, diarrhea, and weight loss. Hypercalcemia has been observed in several case reports [12–16], but it is rarely observed in clinical practice. Radiographic examinations helped assess outcomes [17], with recurrence detected in four of our patients by CT. Most patients were diagnosed by pathology after surgical resection. There was a single report of adenosquamous carcinoma diagnosis by colonoscopy with endoscopic resection pathology [18], and one of seven patients reported adenosquamous carcinoma by colonoscopy preoperatively in our study. However, CT and colonoscopy are of limited value in clarifying the preoperative diagnosis of adenosquamous carcinoma, which may be due to the difficulty of detecting the components of squamous and adenocarcinoma simultaneously.

Our research found a higher proportion of patients (27.3%) with dMMR status than previous data (10–20%) showed for colorectal cancer. Akahoshi reported a patient with PD-L1 overexpression in adenosquamous tissue and systematically higher expression of dMMR in adenosquamous carcinoma of other cancer types [19], which indicated that adenosquamous cancer has a different pathogenesis and tumor environment compared with adenocarcinoma. A study by Hirsch showed that colorectal cancer patients with dMMR responded to immunotherapy [20]. In addition, several articles have also shown that colorectal cancer with dMMR status responded to immunotherapy and Pembrolizumab [21–23]. The aforementioned immunotherapy research targets are basically adenocarcinoma, while few reports have been on CASC because it is an uncommon type of cancer. A report from Evert [24] of successful treatment with pembrolizumab in metastatic CASC provided an excellent therapeutic direction. Deficient MMR protein expression is currently not a routine examination for colorectal patients in many countries. Investigating the proportion of dMMR status might provide variable treatment and management for CASC patients.

We also found that a high proportion of patients exhibited mutations in *KRAS* and that missense mutations occurred at a different codon than in colorectal adenocarcinoma. *KRAS* mutations account for 40% of all genetic mutations in colorectal tumors [25], however, due to the small number of patients, there were no previous reports of mutant *KRAS* gene in CASC [26]. Our finding supplements the involvement of *KRAS* in this aggressive histological sub-type of colon cancer. Similar involvement was mentioned in lung cancer in the literature [27]. Furthermore, one study concluded that a *KRAS* mutation is associated with suppressed Th1/cytotoxic immunity in colorectal cancer [28], which provided a basis for interpreting the association between *KRAS* mutations and colorectal adenosquamous carcinoma. *KRAS*^SG12C^allele-specific inhibitors have been developed due to the need for more precise therapies [29]. Lenkiewicz’s study of adenosquamous carcinoma of the pancreas suggested that mutations in the *KRAS* gene provide a therapeutic target for adenosquamous carcinoma [30], which may provide new directions for the treatment of colorectal adenosquamous carcinoma.

The present study has some limitations. First, this was a single-center study, and although the hospital is one of the largest gastrointestinal specialist hospitals in Asia, the patients are likely not representative of the entire Asian population. Second, the population size was relatively small, which may limit the generalizability of the findings. Lastly, the follow-up period was limited, which may underestimate the long-term oncological behavior and prognosis of adenosquamous carcinoma. Therefore, future multi-center studies with larger sample sizes and longer follow-up periods are needed to validate our findings and provide more comprehensive insights into adenosquamous carcinoma’s clinical characteristics, molecular features, and therapeutic options.

## Conclusions

In conclusion, CASC is a rare cancer with a poor prognosis and nonspecific clinical presentations that is often diagnosed at an advanced stage. Moreover, we identified a high proportion of dMMR status and *KRAS* mutations in our CASC patients, which informs the development of targeted therapies like immunotherapy.

## Data Availability

Data and materials were available by contacting corresponding authors upon reasonable requests.

## Abbreviations

dMMR: mismatch repair-deficient
CASC: colorectal adenosquamous carcinoma
